# Deficiency of innate-like T lymphocytes in chronic obstructive pulmonary disease

**DOI:** 10.1186/s12931-017-0671-1

**Published:** 2017-11-28

**Authors:** Mariann Szabó, Veronika Sárosi, Zoltán Balikó, Kornélia Bodó, Nelli Farkas, Tímea Berki, Péter Engelmann

**Affiliations:** 1 Division of Pulmonology, 1st Department of Internal Medicine, Clinical Center, University of Pécs, Rákóczi u. 2, Pécs, H-7623 Hungary; 2 Department of Immunology and Biotechnology, Clinical Center, University of Pécs, Szigeti u. 12, Pécs, H-7643 Hungary; 3 Department of Bioanalysis, Medical School, University of Pécs, Szigeti u. 12, Pécs, H-7643 Hungary

**Keywords:** COPD, Emphysema, iNKT cells, MAIT cells, CD1d, MR1

## Abstract

**Background:**

Based on the phenotypic and functional characteristics unconventional T-lymphocytes such as invariant natural killer T (iNKT) cells and mucosal-associated invariant T (MAIT) cells link the innate and adaptive immune responses. Up to now data are scarce about their involvement in pulmonary disorders including chronic obstructive pulmonary disease (COPD). This study explores simultaneously the frequencies of iNKT and MAIT cells in the peripheral blood and sputum of stable and exacerbating COPD patients.

**Methods:**

By means of multicolor flow cytometry frequencies of total iNKT and MAIT cells and their subsets were enumerated in peripheral blood and sputum samples of healthy controls, and COPD patients. In addition, gene expression of TCR for iNKT, MAIT cells, and CD1d, MR1 were assessed by qPCR in the study cohorts.

**Results:**

Percentages of total iNKT and MAIT cells were dramatically dropped in blood, and reduced numbers of iNKT cells were observed in the sputum of COPD patients. Furthermore decreased DN and increased CD4+ iNKT subsets, while increased DN and decreased CD8+ MAIT subpopulations were measured in the blood of COPD patients. Reduced invariant TCR mRNA levels in COPD patients had confirmed these previous findings. The mRNA expression of CD1d and MR1 were increased in stable and exacerbating COPD patients; however both molecules were decreased upon antibiotic and systemic steroid treatments.

**Conclusions:**

Our results support the notion that both invariant T-cell populations are affected in COPD. Further detailed analysis of invariant T cells could shed more light into the complex interactions of these lymphocyte groups in COPD pathogenesis.

**Electronic supplementary material:**

The online version of this article (10.1186/s12931-017-0671-1) contains supplementary material, which is available to authorized users.

## Background

Chronic obstructive pulmonary disease (COPD) is the third leading cause of death worldwide. Pathogenesis of COPD is characterized by the persistent airflow limitations and associated with chronic inflammation of the airways [1].

Several mechanisms underlie the symptoms of COPD; however the major ones are the emphysema, bronchiolitis, and mucus hypersecretion [2]. Cigarette smoking is one of the main causes of the disease, but other environmental contaminants (e.g. pesticides), industrial dust and microbial infections (e.g. *Haemophilus influenzae*, *Pseudomonas aeruginosa*, *Mycobacterium tuberculosis, Moraxella catarrhalis*) are also highly responsible in this disease burden. Next to the environmental conditions endogenous mechanisms (e.g. genetic and epigenetic factors) also play inevitable role in the development of COPD. Another favorable concept claims for the autoimmune origin of COPD that idea is based on the dysregulated inflammatory process in this disorder [3].

In the course of COPD chronic airway inflammation is characterized by the massive involvement of activated leukocytes. Neutrophils, eosinophils, macrophages and different subsets of lymphocytes infiltrate the lungs. Inhaled environmental factors trigger the engagements of pattern recognition receptors. Their activation leads to the recruitment of leukocytes through the release of various chemokines, and pro-inflammatory cytokines [4]. These secreted compounds possess important role in the progressive airflow limitations mediated by fibrosis and enhanced inflammation [5].

Adaptive immune cells such as T lymphocytes seem to have a central role in COPD [6]. Recently, different T cell subsets are in the center of intensive research in this disease [4, 7]. Emerging data suggests that innate-like T lymphocytes such as invariant natural killer T (iNKT) cells and mucosal-associated invariant T (MAIT) cells are also involved in the pathogenesis of COPD [8, 9]. iNKT and MAIT cells represent unique, unconventional T cell populations characterized by the co-expression of unique TCR and NK cell receptors. Human iNKT cells express the invariant Vα24-Jα18 TCR α-chain combined with the limited diversity V β11 chain, while human MAIT cells express the distinctive Vα7.2-Jα33 TCR α-chain. Based on the CD4 and CD8 co-receptor expression three iNKT cell subgroups (CD4+, DN, CD8+) can be distinguished. DN iNKT cells produce mainly Th1 cytokines, while CD4+ iNKT cells secrete both Th1 and Th2 cytokines [10]. MAIT cells can be further divided into a minor DN and major CD8+ subpopulations. These innate T-lymphocytes are restricted by non-classical antigen presenting molecules. iNKT cells recognize diverse bacterial glycolipids presented by CD1d, while MAIT cells engaged by microbial vitamin B derivates (e.g. riboflavin, folic acid) presented by the highly conserved non-polymorphic class 1b antigen presenting molecule MHC-1 related protein 1 (MR1) [11, 12].

It has been reported that both subgroups have biased frequencies and functions in several immune-mediated disorders such as autoimmune diseases, and allergy [8, 13–15]. The available data are scarce about the potential role of invariant T subpopulations in the pathogenesis of COPD. So far, more data discuss the role of NKT cells in COPD [9]; however the published results are rather inconsistent in terms of proportional changes of NKT cells in COPD patients. Relatively limited information corresponds to the role of MAIT cells in COPD [16, 17].

Taken this information into account our aim was to characterize simultaneously the proportions and subsets of these two invariant T cell populations in peripheral blood and induced sputum samples of stable COPD and exacerbating COPD (AECOPD) patients.

## Methods

### Patient and control groups

We recruited 38 volunteers: 17 healthy controls (mean age ± SD, 50.6 ± 9.3 years), 11 stable COPD and 10 exacerbating COPD patients into the study. Subjects with any known autoimmune or malignant disease in the last 5 years were excluded from the study. Enrolled COPD patients were characterized according to the international GOLD guidelines: post-bronchodilator FEV_1_/FVC ratio was <70% and mild (GOLD1: FEV_1_ ≥ 80% predicted), moderate (GOLD2: 50% ≤ FEV_1_ < 80% predicted), severe (GOLD3: 30% ≤ FEV_1_ < 50% predicted), very severe (GOLD4: FEV_1_ < 30% predicted) stages were applied as disease categories. All enrolled AECOPD patients were in severe or very severe stages, while stable COPD patients were in moderate, severe and very severe stages. Blood samples were taken as standard procedures in vacuum blood collecting tubes. First blood drawn was executed in the morning at the Division of Pulmonology within 24 h after the admission in the Department of Emergency, University of Pécs, where they received treatments (inhalative β antagonist and corticosteroids). Second blood drawn and sputum induction of AECOPD patients was executed 72 h later of the first blood drawn in the morning at the Division of Pulmonology. Healthy controls and stable COPD patients were the subject of simultaneous blood drawn and sputum induction. COPD patients were treated with standard inhalative medications (LABA, LAMA, ICS + LABA) based on the GOLD guidelines. Exacerbating patients received systemic corticosteroids and antibiotics next to the regular inhalative medications. To uncover any treatment effects the AECOPD patient samples were analyzed separately at the beginning of medications and during the ongoing medications, marked in datasets as AECOPD (a) or AECOPD (b), respectively. Basic clinical characteristics of COPD patients are detailed in Table 1. The study was approved by Regional Research Ethics Committee of the Faculty of Medicine at the University of Pécs (5414/2014) and written informed consent forms were obtained from all donors.

**Table 1:** Characteristics of the COPD patients enrolled in the study

	Stable COPD cohort	AECOPD cohort
Age	60.8 ± 8.99	64.9 ± 14.09
CAT	21.8 ± 8.1	31.4 ± 5.87^*^
CCQ	28.1 ± 15.88	43.2 ± 9.041^*^
FEV_1_ (% predicted)	54.45 ± 12.34	41.8 ± 18.9
FEV_1_ (liter)	1.612 ± 0.558	1.027 ± 0.4942
Tiffenau-index	53.73 ± 7.129	47.43 ± 9.467
Treatments		
Inhaled steroids (Y/N)	6/5	10/0
Long acting muscarinic agonist (LAMA) (Y/N)	11/0	10/0
Long acting β agonist (LABA) (Y/N)	8/3	10/0

Data are presented as mean ± SD and tested by *t*-test (^*^ *p* < 0.05); *CAT* COPD Assessment Test, *CCQ* Clinical COPD Questionnaire, *Y/N* yes or no.

### Sputum induction and processing

Sputum samples were collected from the blood donors. Sputum was induced by inhalation of hypertonic salt aerosol (4% NaCl) generated by a MiniPlus Compressor Nebulizer (Apex Medical Corp., Taipei, Taiwan) according to the ERS guidelines [18, 19]. After the induction sputum was processed within 30 min, an equal volume of 0.1% dithiothreitol solution (DTT, Sigma) was administrated. Samples were mixed with end-over-end rotator and incubated at 37 °C for 15 min to make firm homogenization. The samples were then diluted with PBS and filtered through a 50-μm pore size cell strainer. Filtered samples were centrifuged at 1000 rpm for 5 min and then cell viability was assessed by trypan blue exclusion test. Samples were further processed for flow cytometry measurements.

### Flow cytometry

Peripheral blood and processed sputum samples of healthy controls and COPD patients were stained with various fluorochrome-coupled mouse monoclonal antibodies specific for human leukocyte surface markers: anti-Vα24-Jα18-FITC, a-Vα7.2-FITC, a-CD8-PE, a-CD4-PerCPCy5.5, a-CD161-PerCPCy5.5, a-CD3-APC (Biolegend, San Diego, CA, USA). After incubation erythrocytes were lysed with FACS Lysing Solution (BD Biosciences). Flow cytometry data were acquired with BD FACSCalibur flow cytometer and the analysis were performed with FCS Express 4 software (De Novo Software, Glendale, CA, USA).

### RNA isolation and cDNA synthesis

Peripheral blood mononuclear cells (PBMCs) were obtained from fresh blood of healthy individuals and COPD patients by Ficoll (Amersham Pharmacia Biotech Europe, Uppsala, Sweden) gradient centrifugation following standard protocol. Isolated PBMCs were resuspended and frozen in RNALater solution (Ambion, Thermo Fisher Scientific, Waltham, MA, USA) and stored according to the manufacturer’s instructions until sample processing.

RNA extraction was performed using NucleoSpin® RNA isolation kit (Macherey-Nagel GmbH, Düren, Germany) according to the manufacturer’s guidelines (including DNase I digestion). Isolated RNA was re-suspended in nuclease free water, quantified at 260 nm and the quality of total RNA was confirmed by 1% agarose gel analysis.

The cDNA was constructed from total RNA with High Capacity cDNA reverse transcription kit (Thermo Fisher Scientific) in 20 μl reactions using random hexamers following the manufacturer’s protocol. The resulting cDNA was stored at −20 °C.

### Quantitative real-time PCR

Target gene expression was measured by real-time PCR using Maxima SYBRGreen MasterMix (Applied Biosystems) with an ABI Prism 7500 instrument (Applied Biosystems). The PBMC cDNAs were used as a template for the amplification reactions. All samples were tested in duplicates. Primers were designed using Primer Express software (Thermo Fisher Scientific) considering the exon-intron boundaries for all target genes. In the case of invariant TCR α chain primers were designed around the flanking of Variable, Joining and Constant rearrangement regions (Table 2). Thermal profile started at 95 °C for 10 min, 40 cycles of 35 s at 95 °C, 35 s at 60 °C, 1 min at 72 °C.

**Table 2:** Primer sequences applied for qPCR

Target gene	GenBank Accession#	Primers (5′-3′)^a^	Amplicon size (bp)
TATA-binding protein (TBP)	BC110341	CCA GAC TGG CAG CAA GAA AAT TCA CAG CTC CCC ACC ATA TTC	100
Vα24-Jα18(iNKT) TCR	DQ341448	AAG ATA TAC AGC AAC TCT GGA TGC A CTG TCG CTC ACC ACA CAG ATG	105
Vα7.2-Jα33(MAIT) TCR	EU885186	GTG CTG TGA AGG ATG GCA ACT CGG CAG GGT CAG GGT TCT	90
CD1d	NM_001766	CGC TGA AGT CCC GCA AAG GCT ATT GGC GAA GGA CGA GAT	64
MR1	BC012485	AGG CCC CGA GAG CAA AAT AGG ATA ACA TGG CTC CTA GAG GAA	102

^a^Upper and lower sequences represent forward and reverse primers, respectively

### Statistical analysis

Statistical analysis was performed with GraphPad Prism version 5 (GraphPad Software Inc., La Jolla, CA, USA). Variables were expressed as medians and all whiskers represent 1.5 interquartile range. In the case of non-Gaussian distribution the effect of treatments were analyzed by Kruskal-Wallis-test. One way-ANOVA analysis was performed on the data with normal distributions. Both analyses were followed by Dunn’s or Bonferroni post hoc tests, respectively. *p* < 0.05 was denoted as statistically significant.

## Results

### Decreased frequency of total iNKT cells and biased proportions of iNKT subgroups in stable COPD and AECOPD patients

**Fig. 1:**
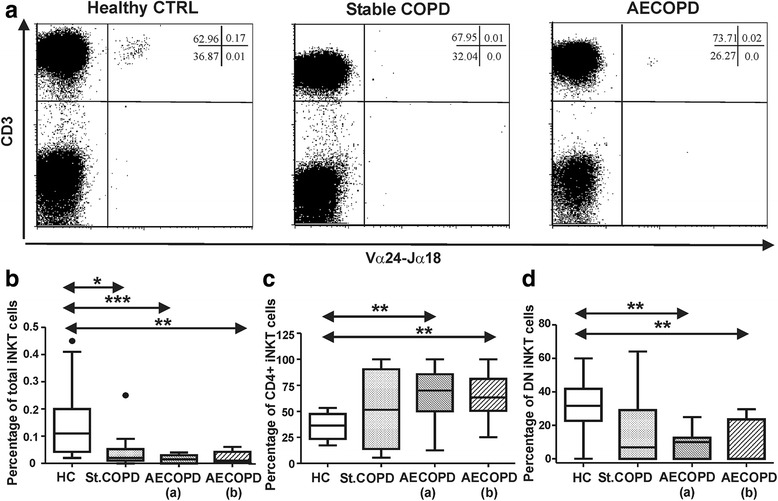
Peripheral blood iNKT cells of healthy controls (HC), stable COPD and exacerbating COPD (AECOPD) patients. **a** Representative flow cytometry dot-plots of iNKT cells stained with Vα24-Jα18 and CD3 specific antibodies. **b** Decreased total iNKT populations were detected in stable COPD and AECOPD patients. **c** Significantly elevated CD4+ iNKT subpopulation were enumerated in AECOPD patients compared to the controls. **d** Significantly decreased DN iNKT subsets were observed in AECOPD patients. Percentages were calculated after gating on CD3+ and Vα24-Jα18+ iNKT cells. Data present here were derived from seventeen HC, eleven stable COPD and ten AECOPD blood donors. Boxes show interquartile ranges (IQR), whiskers represent lowest and highest values, horizontal lines indicate median, and circles stand for outliers of 1.5 IQR. Asterisks represent significant *p* (* < 0.05, ** < 0.01, *** < 0.001) values

iNKT cells are rare lymphoid cells in human peripheral blood. We compared the overall proportions of iNKT cells in the blood samples of healthy controls, stable COPD and AECOPD patients by applying multicolor flow cytometry (Fig. 1a). After gating on lymphocytes iNKT cells were defined according to the staining with anti-Vα24-Jα18-FITC and a-CD3-APC antibodies. iNKT subsets were characterized by a-CD8-PE and a-CD4-PerCPCy5.5 antibodies (Additional file 1: Figure S1A). We assessed that whether the effects of the systemic steroid and antibiotic treatments compromise the proportions of iNKT cell subsets. We found significant decrease in the total iNKT population of stable COPD and AECOPD patients compared to healthy controls (Fig. 1a and b). Furthermore, we observed biased frequencies of CD4+ and DN iNKT subpopulations, since CD4+ iNKT cells were elevated (Fig. 1c), while DN iNKT subpopulation had a significant decrease in the AECOPD patients compared to healthy controls (Fig. 1d). Minor CD8+ iNKT population did not indicate any characteristic changes (data not shown). There was no significant difference between the iNKT cells of stable COPD and AECOPD patient groups. Regarding to smoker and non-smoker (non-recent smoker individuals who stopped smoking minimum two years before sample collection) status we observed significant differences in the iNKT population of stable COPD patients. Smoker patients have significantly reduced iNKT cell frequencies compared to non-recent smokers (Additional file 2: Figure S2A). In addition we compared the frequencies of total iNKT cells from smoker HC, stable COPD and AECOPD individuals. We found significant decrease in iNKT proportions of AECOPD patients compared to HC cohort, while iNKT cells from smoker stable COPD patients evidenced a non-significant decrease compared to the smoker HC group (Additional file 2: Figure S2C). Smoking-related information of healthy subjects and COPD patients are detailed in Table 3.

**Table 3:** Smoking-related information of healthy subjects and COPD patients enrolled in the study

	Healthy ctrl cohort	Stable COPD cohort	AECOPD cohort
Gender (F/M)	8/9	3/8	3/7
Smoking (never/earlier/current)	11/1/5	0/6/5	0/5/5
Packs/years	24.3 ± 22.27	30.05 ± 13.22	38.47 ± 28.31
Smoking cessation	> 2 years	> 2 years	> 2 years

Packs/years data are presented as mean ± SD

### Decreased proportions of total MAIT cells and biased DN, CD8+ MAIT subpopulations in stable COPD and AECOPD patients

**Fig. 2:**
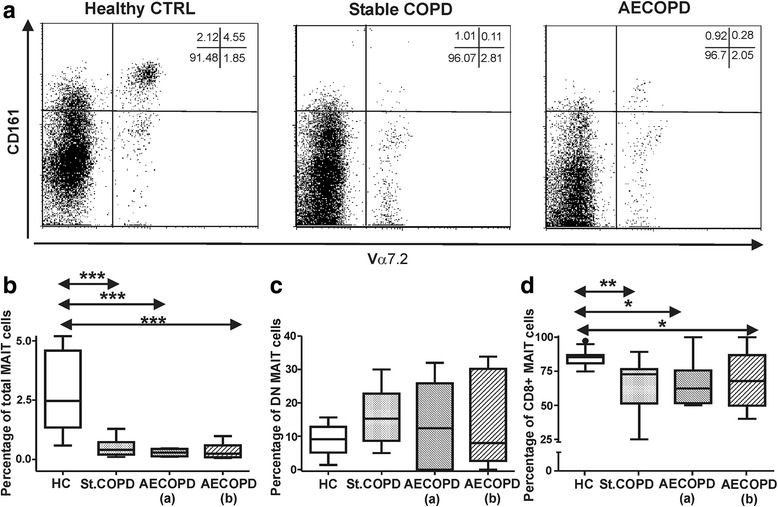
Peripheral blood MAIT cells of healthy controls (HC), stable COPD and AECOPD patients. **a** Representative flow cytometry dot-plots of MAIT cells stained with Vα7.2 and CD161 specific antibodies after gating on CD3+ cells. **b** Significantly decreased MAIT cells were detected in stable COPD and AECOPD patients. **c** Non-significant increase of DN MAIT subsets was observed in COPD patients compared to the healthy controls. **d** Significant decrease of CD8+ MAIT subpopulation was enumerated in the peripheral blood of COPD patients. Percentages were calculated after gating on CD3+ T cells. Data present here were derived from seventeen HC, eleven stable COPD and ten AECOPD blood donors. Boxes show interquartile ranges (IQR), whiskers represent lowest and highest values, horizontal lines indicate median, and circles stand for outliers of 1.5 IQR. Asterisks represent significant *p* (* < 0.05, ** < 0.01, *** < 0.001) values

After gating on CD3+ lymphocytes MAIT cells were defined by staining with anti-Vα7.2-FITC and a-CD161-PerCPCy5.5 antibodies. DN and CD8+ MAIT cell subsets were characterized by a-CD8-PE staining (Additional file 1: Figure S1B). Indeed, MAIT cells are more frequent in human peripheral blood compared to iNKT cells. Measured iNKT cell percentages were exclusively below 0.5%, while MAIT cells evidenced 2.5% high frequencies in the blood samples of healthy controls (Figs. 1 and 2). Similarly to iNKT cells we observed significantly dropped MAIT cell numbers in the peripheral blood of stable COPD and AECOPD patients compared to healthy controls (Fig. 2a and b). We found non-significant increase of DN MAIT subpopulation compared to the controls (Fig. 2c). Furthermore, the proportions of CD8+ MAIT cells were significantly decreased in COPD patient groups compared to healthy controls (Fig. 2d). There was no difference of total and DN or CD8+ MAIT cells between stable COPD and AECOPD patients. Besides, we measured significantly decreased total MAIT cell proportions in smoker, stable COPD patients compared to the non-recent smoker populations (Additional file 2: Figure S2B). In addition we compared the frequencies of total MAIT cells from smoker HC, stable COPD and AECOPD individuals. We found significant decrease in MAIT cell proportions of both smoker stable and AECOPD patients compared to smoker HC cohort (Additional file 2: Figure S2D).

Smoker, stable COPD patients have significantly increased DN and decreased CD8+ MAIT subpopulations (Additional file 3: Figure S3A and B). In smoker AECOPD population DN MAIT cells were non-significantly increased, while CD8+ MAIT cells were significantly reduced compared to the non-smoker AECOPD population (Additional file 3: Figure S3C and D). We did not find any proportional difference in MAIT cells between the smoker or non-smoker healthy populations (Additional file 4: Figure S4B).

### Decreased iNKT and MAIT cell proportions in the induced sputum of COPD patients

**Fig. 3:**
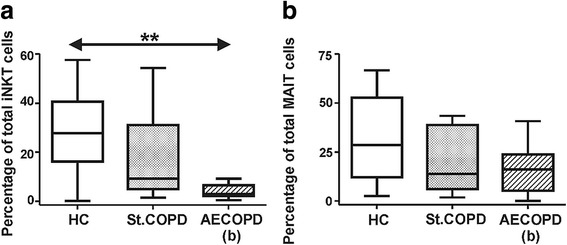
Frequencies of iNKT (**a**) and MAIT (**b**) cells in the induced sputum of healthy controls (HC), stable COPD and AECOPD patients. **a** Decreased iNKT populations were observed in the stable COPD and AECOPD patients. **b** MAIT cells have evidenced a non-significant decrease in the sputum of stable COPD and AECOPD patients. Percentages were calculated after gating on CD3+ T cells. Data present here were derived from fifteen HC, six stable COPD and seven AECOPD donors (please note that several sputum inductions were unsuccessful to isolate mononuclear cells for flow cytometry analysis). Boxes show interquartile ranges (IQR), whiskers represent lowest and highest values, horizontal lines indicate median. Asterisks represent significant *p* (** < 0.01) values

We aimed to assess whether the invariant T cells are differently represented in the airways compared to the peripheral blood. Applying the same staining set-up we measured the frequencies of iNKT and MAIT cells in the induced sputum of healthy subjects and COPD patients. Albeit the frequencies of iNKT cells were higher among the lymphocytes in the sputum than in peripheral blood, but we found a significant decrease of this invariant T cell population in the AECOPD patients compared to the healthy controls (Fig. 3a). iNKT cells evidenced non-significant decrease in the stable COPD patients.

In the case of sputum MAIT cells we have not found differences between healthy controls and COPD patients; however we observed a non-significant trend of decrease in MAIT cell proportions in stable COPD and AECOPD patients compared to the control population (Fig. 3b).

### Decreased mRNA expression profile of the iNKT and MAIT TCRs in COPD patients

**Fig. 4:**
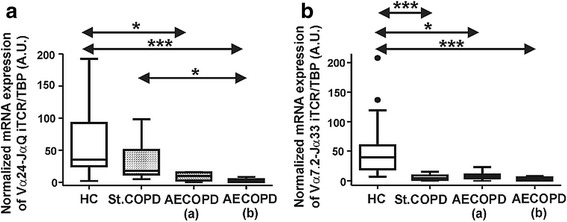
Gene expression levels of iNKT (**a**) and MAIT (**b**) cell-restricted invariant TCRs in healthy controls (HC), stable COPD and AECOPD patients. **a** iNKT-cell specific Vα24-Jα18 (JαQ) TCR gene expression levels were decreased in COPD patients. **b** MAIT-cell specific Vα7.2-Jα33 TCR gene expression was reduced in stable COPD and AECOPD patients. Data present here were derived from sixteen HC, ten stable COPD and ten AECOPD blood donors. Boxes show interquartile ranges (IQR), whiskers represent lowest and highest values, horizontal lines indicate median, and circles stand for outliers of 1.5 IQR. Asterisks represent significant *p* (* < 0.05, *** < 0.001) values. A.U.: arbitrary units

Besides the flow cytometry analysis we assessed the mRNA expression levels of the canonical invariant TCR in iNKT and MAIT cells. Being in agreement with the flow cytometry-based observations we measured significant decrease of the Vα24-Jα18 invariant alpha chain in iNKT cells of AECOPD patients compared to the healthy controls. Interestingly, we observed significant difference between stable COPD and AECOPD (b) patients in the mRNA levels of iNKT TCR (Fig. 4a).

Furthermore, we evaluated the expression levels of Vα7.2 MAIT TCR in the aforementioned study groups. Significantly decreased expression pattern of Vα7.2-Jα33 TCR mRNA message was determined in all COPD patients compared to the healthy controls (Fig. 4b).

### Biased mRNA expression patterns of CD1d and MR1 antigen presenting molecules in COPD patients

**Fig. 5:**
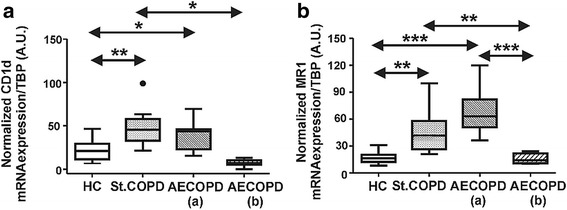
Gene expression patterns of CD1d (**a**) and MR1 (**b**) antigen presenting molecules in healthy controls (HC), stable COPD and AECOPD patients. **a** Significantly elevated CD1d gene expression was observed in stable COPD and AECOPD patient groups at the beginning of systemic steroid and antibiotic treatments. **b** Significantly elevated MR1 gene expression was assessed in stable COPD and AECOPD patients at the beginning of systemic steroid and antibiotic treatments. Data present here were derived from sixteen HC, ten stable COPD and ten AECOPD blood donors. Boxes show interquartile ranges (IQR), whiskers represent lowest and highest values, horizontal lines indicate median, and circles stand for outliers of 1.5 IQR. Asterisks represent significant *p* (* < 0.05, ** < 0.01, *** < 0.001) values. A.U.: arbitrary units

Next we assessed the CD1d and MR1 mRNA expression levels. We found that CD1d expression is significantly increased in stable COPD and AECOPD (a) patients compared to the healthy controls. In contrast, upon treatment the CD1d expression of AECOPD (b) patients was not significantly different from the healthy control group; however it was significantly decreased compared to stable COPD patients (Fig. 5a).

Moreover we checked the expression levels of the MAIT cell-engaged MR1 molecules in the study groups. Interestingly, we found that MR1 expression levels were significantly increased in stable COPD and AECOPD (a) patients, while there was no significant change of MR1 expression in the treated hospitalized AECOPD (b) patients compared to the healthy controls (Fig. 5b).

## Discussion

Exposure to cigarette smoke initiates a chronic inflammatory response hallmarked by cellular infiltration in COPD [20]. It is suggested that the proportions of neutrophils are inversely correlated with the extension of emphysema, while the amount of infiltrating macrophages and T cells are positively correlated with the level of lung destruction. In the course of COPD pathogenesis involved T cell subgroups and their pathogenic role are just partly uncovered [3]. Recent information claims that a large number of T cells reside in the inflamed lung and mostly CD8+ T cells are responsible for the development of emphysema [21].

In this attempt we undertake the task to characterize and enumerate the distinct innate-like T lymphocyte subsets (namely invariant NKT and MAIT cells) in the peripheral blood and sputum samples of COPD patients. It is necessary to note that NKT cells are phenotypically and functionally diverse lymphocytes. These innate-like T cells contain the extensively characterized CD1d-restricted invariant NKT cells (type I or iNKT cells), CD1d-restricted non-invariant (type II) and CD1d independent (type III) NKT cells [22]. In fact, the role of NKT cells in pulmonary disorders is quite controversial that could be due to their diversity and the inconsistent application of diagnostic reagents to identify them [9]. All these obstacles detain the objective comparisons of independent experimental results. Furthermore, several line of evidence proved that the aforementioned type I and type II NKT cells frequently unfold opposing effects on each other in certain pathological conditions [23, 24].

There are several staining procedures to analyze NKT cells, but among those only the Vα24-Jα18 / CD3 or αGalCer (exogenous model antigen)-loaded CD1d tetramer / CD3 co-staining seems reliable to identify equivocally the iNKT cells [25, 26].

Based on the Vα24-Jα18 / CD3 co-staining our results are partly concordant with the previous findings [27] concerning decreased iNKT cells in stable COPD and exacerbating COPD patients. We measured a significant drop of total iNKT cells in the peripheral blood of COPD patients compared to healthy subjects. Furthermore our qPCR analysis confirmed these findings and supported the notion that total iNKT population was decreased in COPD. Indeed, in the aforementioned study [27] several different staining patterns have been performed to analyze iNKT cells in the peripheral blood of COPD patients. According to our flow cytometry and qPCR results we can conclude that blood iNKT cell numbers are decreased in COPD patients.

On the other hand it has been reported that CD4+ iNKT subset was decreased in stable COPD and AECOPD patients [27]. In contrast, we found that the frequencies of CD4+ iNKT cells have been significantly increased in the peripheral blood of AECOPD patients and non-significantly elevated in the peripheral blood of stable COPD patients. Other iNKT cell subsets (DN, CD8+) have not been enumerated [27]. In our AECOPD study cohort the DN iNKT population evidenced decreased proportions, while CD8+ iNKT cells did not demonstrate any difference compared to the healthy controls. Conversely to the aforementioned publication [27] we did not observe significant differences of circulating iNKT cells in stable COPD and AECOPD patients. To the best of our knowledge, there is no available publication about the distribution of the DN and CD8+ iNKT cell subsets in COPD patients.

By the means of CD3 / CD56 co-staining classifies the mixed population of NKT-like lymphocytes consist of iNKT, type II NKT cells and other T cells. Therefore the measured frequencies of NKT-like cells can be falsely interpreted due to their complexity [28]. By means of CD3 / CD56 double-labeling several publications reported biased proportions of NKT-like cells in COPD patients [29–33]. Indeed, these datasets should be handled cautiously according to the previously described considerations.

All aforementioned publications reported about elevated proportions of NKT-like cells in the sputum of COPD patients. In contrast we observed decreased iNKT cell frequencies in the sputum of COPD patients compared to controls similarly to those measured in peripheral blood. Indeed, an explanation for the discrepancy among our data and others could be the different staining methods and detection of mixed populations of NKT-like cells [31].

One additional study enumerated low amounts of iNKT cells in bronchoalveolar lavage and sputum samples of control, allergic and COPD patients [34]. There was no significant difference among the iNKT proportions isolated from controls and COPD patients. Indeed, in this study iNKT cells and not NKT-like cells were analyzed, however the staining combination was different from our approach applying specific antibodies against both α and β chain of the iTCR (Vα24-Jα18 and Vβ11, respectively). This data was verified by qPCR analysis targeting the invariant TCR. Another study concordantly found elevated number of iNKT cells and NKT-like cells in the sputum of COPD patients and in the cigarette smoke induced mouse COPD model [31]. In this report human iNKT cells were identified based on the αGalCer-derivative loaded CD1d tetramer staining. According to Chi et al. [27] αGalCer-loaded CD1d tetramer enhanced the specificity but also increased the variations and could not be observed differences between blood iNKT from COPD patients and healthy controls applying this staining approach; however these authors did not measure the proportions of iNKT cells in the sputum.

So far the identification of MAIT cells is more straightforward compared to those of NKT cells. Since the available antibody reagents such as a-Vα7.2, a-CD3 and a-CD161 only determines one specific staining algorithm to identify specifically MAIT cells. According to the available information MAIT cells are decreased in the peripheral blood of COPD patients compared to controls [17]. In this report decreased proportions of DN and CD8+ MAIT cell subpopulations were evaluated in the COPD study cohorts. Indeed, we found a significant drop of total MAIT cells frequencies in COPD patients. Contrary to the aforementioned observation [17] the proportions of CD8+ MAIT cells were significantly decreased, however the DN MAIT cell population showed a trend for non-significant increase in COPD. Furthermore we evaluated Vα7.2-Jα33 TCR mRNA by qPCR in the control and patient cohorts. We independently found similar decrease of MAIT TCR message in the COPD patients.

MAIT cells showed a slight non-significant decrease in the sputum of COPD patients that is concordant with others findings [16]. This particular study emphasizes that ICS treatments in COPD impaired MAIT cell numbers. In fact, due to limited number of studies we cannot rule out the ICS effect on MAIT cells in COPD however, in our study nearly half of stable COPD patients did not receive ICS treatments and evidenced declined frequencies of MAIT cells and decreased Vα7.2-Jα33 TCR mRNA. MAIT cell frequencies were not significantly different between ICS treated or non-treated stable COPD patients and those from AECOPD patients under strict ICS therapy (Additional file 4: Figure S4A). In addition a novel observation claims that exposure to cigarette smoke can reduce CD8+ MAIT cells in healthy individuals and MS patients [35]. In contrast, we have compared the total MAIT cells (and subsets) of our non-smoker vs. smoker healthy cohorts, but we did not find any significant differences (Additional file 4: Figure S4B).

Unique highly conserved antigen-presenting molecules such as CD1d and MR1 for iNKT and MAIT cells have a broad tissue and cellular expression patterns in human. CD1d is most typically expressed on various hematopoetic antigen presenting cells (APCs, eg. dendritic cells, macrophages, B cells, DP thymocytes) and intestinal epithelial cells, while MR1 is expressed in a wide variety of tissues including lung, liver, spleen, thymus and small intestine, colon and peripheral blood leukocytes [36]. The knowledge about the changes of these antigen presenting molecules is lacking in COPD pathogenesis. Human alveolar macrophages have evidenced a decreased expression of HLA-DR molecules in COPD patients, however there was no such difference in peripheral mononuclear cells [37]. Recently the MR1 expression of in vitro cultured pulmonary macrophages has been enumerated upon incubation with nontypeable *Haemophilus influenzae* (NTHi)*.* MR1 was up-regulated in the NTHi exposed macrophages; however fluticazone and budesonide treatments decreased the MR1 level in the NTHi exposed cells [16]. In our data-set elevated expression of CD1d and MR1 mRNA were measured in stable and AECOPD patients, while the systemic steroid treated AECOPD patients evidenced a dropped pattern for both non-polymorphic antigen-presenting molecules similarly to the previous in vitro results [16]. These results could be explained by the activation status of the APCs. On the other hand it is possible that the APCs try to compensate the loss of stimulatory signals due to the decreased amount of innate-like T cells by the over-expression of these unique antigen presenting molecules.

## Conclusions

To best of our knowledge first we examined simultaneously the proportions of iNKT and MAIT cells in the same COPD patient groups. In concert, with other studies we found decreased invariant T cell numbers in the peripheral blood of COPD patients, whether this is due to ICS treatments or directly related to disease pathogenesis needs to further investigated. In contrast to most studies, we observed all subpopulations of iNKT and MAIT cells. These subpopulations have unique functional differences that might correspond to the disease progression. Furthermore we evaluated the expression of iNKT and MAIT iTCR and CD1d/MR1 molecules that evidenced characteristic bias in COPD. Indeed, a better understanding of cellular interactions between invariant T cells and APCs might lead for future cellular-based therapies in COPD [38, 39].

## Additional files

Additional file 1: Figure S1. Representative flow cytometry dot plots reveal the gating strategy to identify iNKT cells and their DN, CD4+, CD8+ subsets (**A**). Representative flow cytometry dot plots and a histogram demonstrate the gating strategy to identify MAIT cells and their DN, CD8+ subsets (**B**). (PDF 199 kb)

Additional file 2: Figure S2. Percentages of total iNKT (**A**) and MAIT (**B**) cells were enumerated in the peripheral blood of non-smoker and smoker populations in stable COPD patient cohort. Data present here were derived from six non-smoker and five stable COPD blood donors. Percentages of total iNKT (**C**) and MAIT (**D**) cells were enumerated in the peripheral blood of smoker populations in HC, stable COPD and AECOPD patient cohorts. Data present here were derived from five-five smoker HC, smoker stable COPD and smoker AECOPD blood donors. Boxes show interquartile ranges (IQR) whiskers represent lowest and highest values, horizontal lines indicate median. (PDF 158 kb)

Additional file 3: Figure S3. Percentages of DN (**A**) and CD8+ (**B**) MAIT cells were evaluated in the peripheral blood of non-smoker and smoker populations in stable COPD patient cohort. Data present here were derived from six non-smoker and five smoker stable COPD blood donors. Percentages of DN (**C**) and CD8+ (**D**) MAIT cells were measured in the peripheral blood of non-smoker and smoker populations in the AECOPD patient cohort. Data present here were derived from five non-smoker and five smoker AECOPD blood donors. Boxes show interquartile ranges (IQR) whiskers represent lowest and highest values, horizontal lines indicate median. Asterisks represent significant *p* (* < 0.05) values. (PDF 129 kb)

Additional file 4: Figure S4. Percentages of total MAIT cells were evaluated in the peripheral blood of inhaled corticosteroid treated (ICS) or non-treated (No ICS) stable COPD and AECOPD patients (**A**). Data present here were derived from five/six stable COPD patients under no ICS/ICS therapy, and ten AECOPD blood donors all receiving ICS therapy. Percentages of total MAIT cells were measured in the peripheral blood of non-smoker and smoker populations of healthy controls (**B**). Data present here were derived from eleven non-smoker and five smoker healthy control blood donors. Boxes show interquartile ranges (IQR) whiskers represent lowest and highest values, horizontal lines indicate median. (PDF 65 kb)

## Abbreviations

AECOPD: Acute exacerbating chronic obstructive pulmonary disease
APC: Antigen presenting cell
COPD: Chronic obstructive pulmonary disease
DTT: Dithiothreitol
HLA: Human leukocyte antigen
ICS: Inhalative corticosteroids
iNKT: Invariant natural killer T cells
iTCR: Invariant T-cell receptor
LABA: Long acting β agonist
LAMA: Long acting muscarinic agonist
MAIT: Mucosal-associated invariant T cells
MS: Multiple sclerosis
NTHi: Nontypeable *Haemophilus influenzae*
PBS: Phosphate buffered saline
