# Supplementary material for: Deficiency of innate-like T lymphocytes in chronic obstructive pulmonary disease
Source: Respir Res. 2017 Nov 28;18:197. doi: 10.1186/s12931-017-0671-1 (PMC5704534; doi:10.1186/s12931-017-0671-1)
Supplement: Supplementary file 1 — Representative flow cytometry dot plots reveal the gating strategy to identify iNKT cells and their DN, CD4+, CD8+ subsets (A). Representative flow cytometry dot plots and a histogram demonstrate the gating strategy to identify MAIT cells and their DN, CD8+ subsets (B). (PDF 199 kb) [file 12931_2017_671_MOESM1_ESM.pdf]

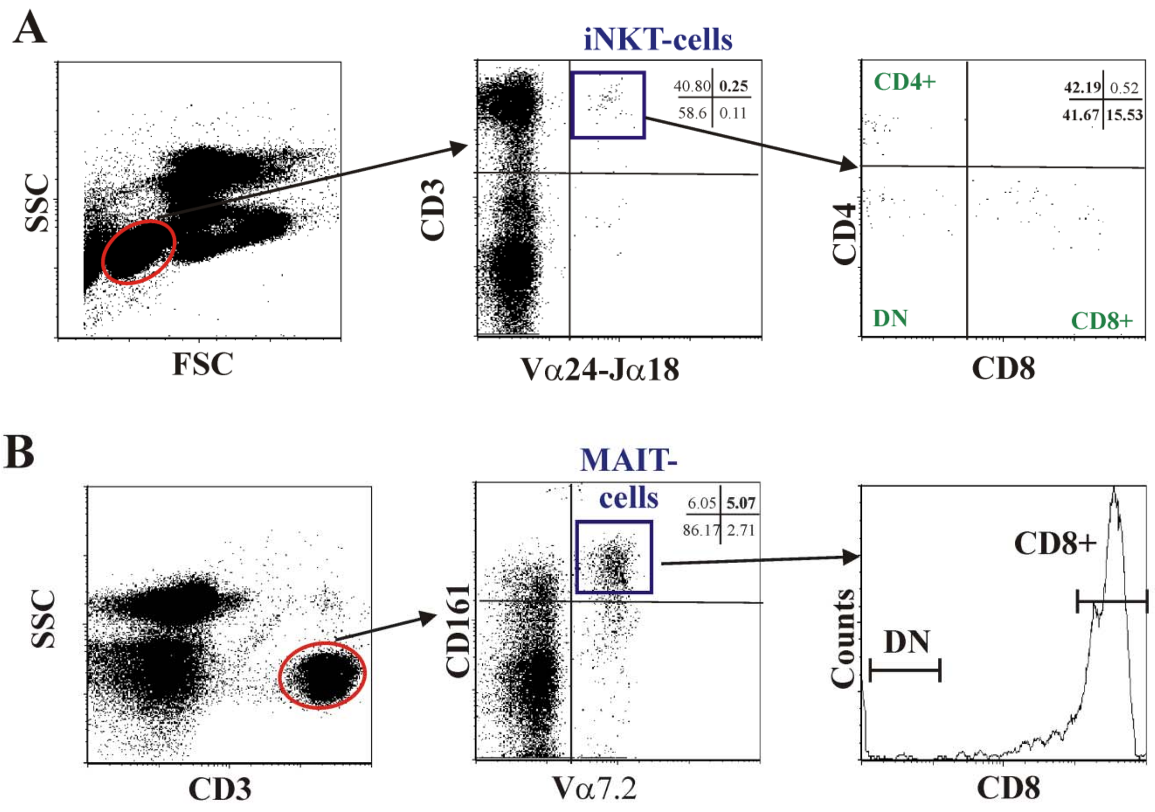

Fig. S1. Representative flow cytometry dot plots reveal the gating strategy to identify iNKT cells and their DN, CD4+, CD8+ subsets (**A**). Representative flow cytometry dot plots and a histogram demonstrate the gating strategy to identify MAIT cells and their DN, CD8+ subsets (**B**).
