# Supplementary material for: Deficiency of innate-like T lymphocytes in chronic obstructive pulmonary disease
Source: Respir Res. 2017 Nov 28;18:197. doi: 10.1186/s12931-017-0671-1 (PMC5704534; doi:10.1186/s12931-017-0671-1)
Supplement: Supplementary file 2 — Percentages of total iNKT (A) and MAIT (B) cells were enumerated in the peripheral blood of non-smoker and smoker populations in stable COPD patient cohort. Data present here were derived from six non-smoker and five stable COPD blood donors. Percentages of total iNKT (C) and MAIT (D) cells were enumerated in the peripheral blood of smoker populations in HC, stable COPD and AECOPD patient cohorts. Data present here were derived from five-five smoker HC, smoker stable COPD and smoker AECOPD blood donors. Boxes show interquartile ranges (IQR) whiskers represent lowest and highest values, horizontal lines indicate median. (PDF 158 kb) [file 12931_2017_671_MOESM2_ESM.pdf]

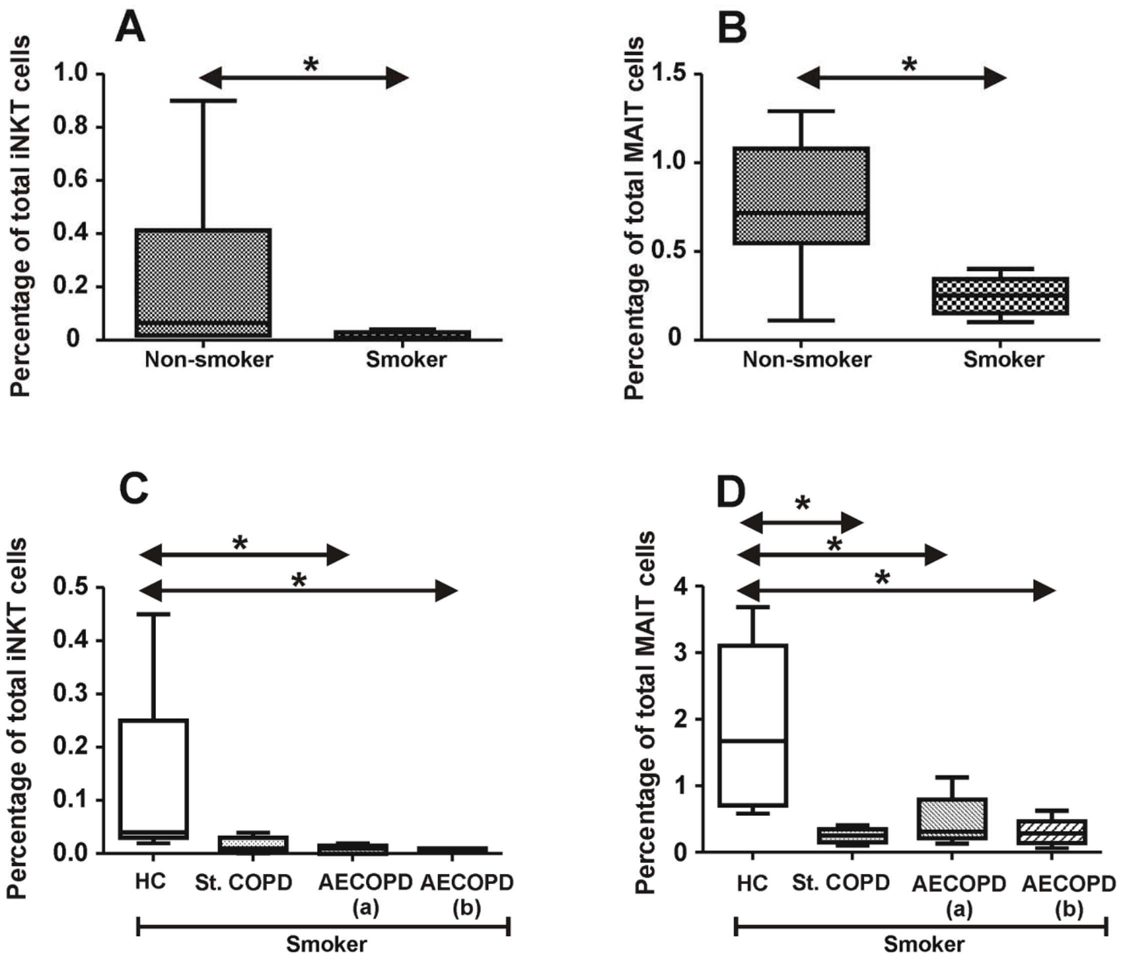

Fig. S2. Percentages of total iNKT (**A**) and MAIT (**B**) cells were enumerated in the peripheral blood of non-smoker and smoker populations in stable COPD patient cohort. Data present here were derived from six non-smoker and five stable COPD blood donors. Percentages of total iNKT (**C**) and MAIT (**D**) cells were enumerated in the peripheral blood of smoker populations in HC, stable COPD and AECOPD patient cohorts. Data present here were derived from five-five smoker HC, smoker stable COPD and smoker AECOPD blood donors. Boxes show interquartile ranges (IQR) whiskers represent lowest and highest values, horizontal lines indicate median.
