# Supplementary material for: Deficiency of innate-like T lymphocytes in chronic obstructive pulmonary disease
Source: Respir Res. 2017 Nov 28;18:197. doi: 10.1186/s12931-017-0671-1 (PMC5704534; doi:10.1186/s12931-017-0671-1)
Supplement: Supplementary file 3 — Percentages of DN (A) and CD8+ (B) MAIT cells were evaluated in the peripheral blood of non-smoker and smoker populations in stable COPD patient cohort. Data present here were derived from six non-smoker and five smoker stable COPD blood donors. Percentages of DN (C) and CD8+ (D) MAIT cells were measured in the peripheral blood of non-smoker and smoker populations in the AECOPD patient cohort. Data present here were derived from five non-smoker and five smoker AECOPD blood donors. Boxes show interquartile ranges (IQR) whiskers represent lowest and highest values, horizontal lines indicate median. Asterisks represent significant p (* < 0.05) values. (PDF 129 kb) [file 12931_2017_671_MOESM3_ESM.pdf]

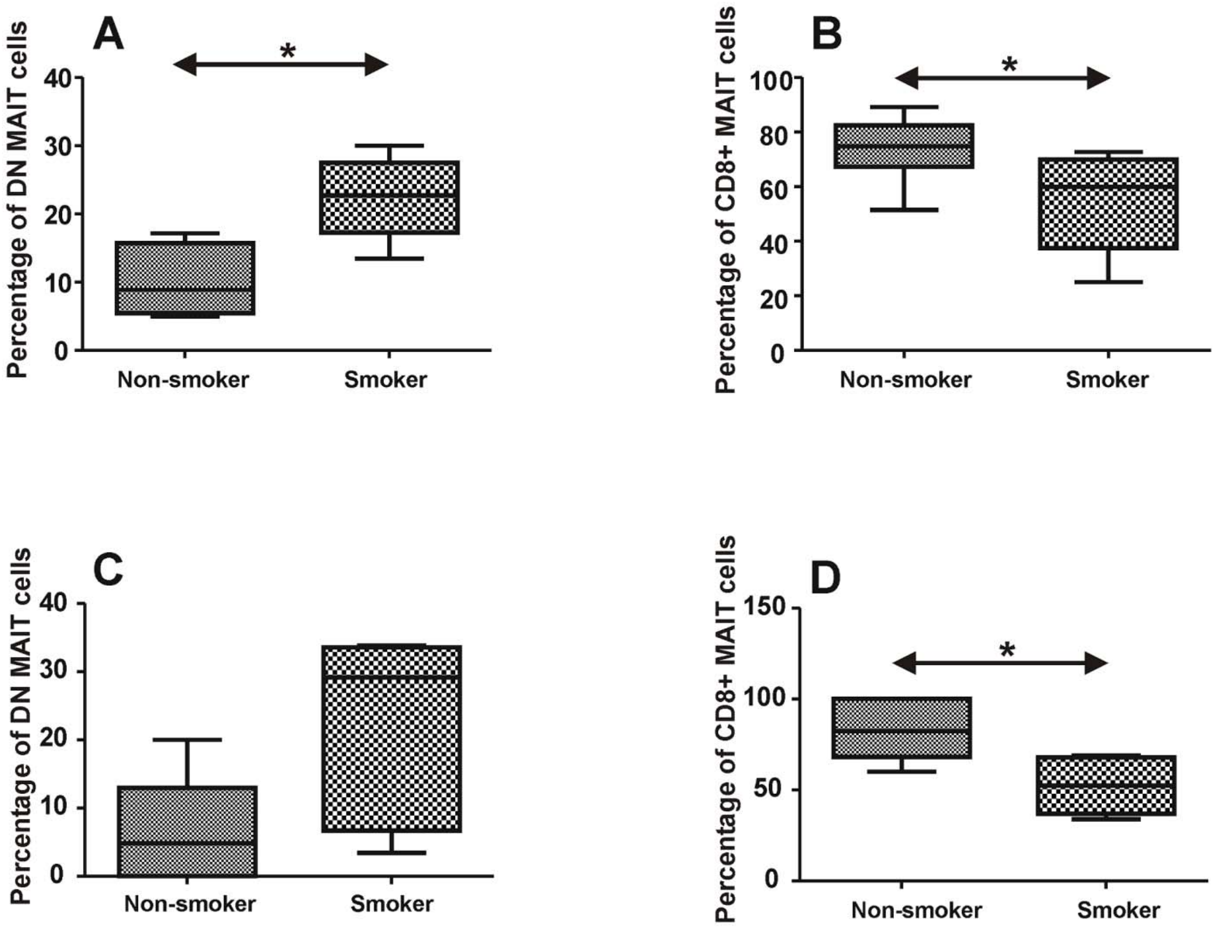

Fig. S3. Percentages of DN (**A**) and CD8+ (**B**) MAIT cells were evaluated in the peripheral blood of non-smoker and smoker populations in stable COPD patient cohort. Data present here were derived from six non-smoker and five smoker stable COPD blood donors. Percentages of DN (**C**) and CD8+ (**D**) MAIT cells were measured in the peripheral blood of non-smoker and smoker populations in the AECOPD patient cohort. Data present here were derived from five non-smoker and five smoker AECOPD blood donors. Boxes show interquartile ranges (IQR) whiskers represent lowest and highest values, horizontal lines indicate median. Asterisks represent significant p (*< 0.05) values.
