# Supplementary material for: Deficiency of innate-like T lymphocytes in chronic obstructive pulmonary disease
Source: Respir Res. 2017 Nov 28;18:197. doi: 10.1186/s12931-017-0671-1 (PMC5704534; doi:10.1186/s12931-017-0671-1)
Supplement: Supplementary file 4 — Percentages of total MAIT cells were evaluated in the peripheral blood of inhaled corticosteroid treated (ICS) or non-treated (No ICS) stable COPD and AECOPD patients (A). Data present here were derived from five/six stable COPD patients under no ICS/ICS therapy, and ten AECOPD blood donors all receiving ICS therapy. Percentages of total MAIT cells were measured in the peripheral blood of non-smoker and smoker populations of healthy controls (B). Data present here were derived from eleven non-smoker and five smoker healthy control blood donors. Boxes show interquartile ranges (IQR) whiskers represent lowest and highest values, horizontal lines indicate median. (PDF 65 kb) [file 12931_2017_671_MOESM4_ESM.pdf]

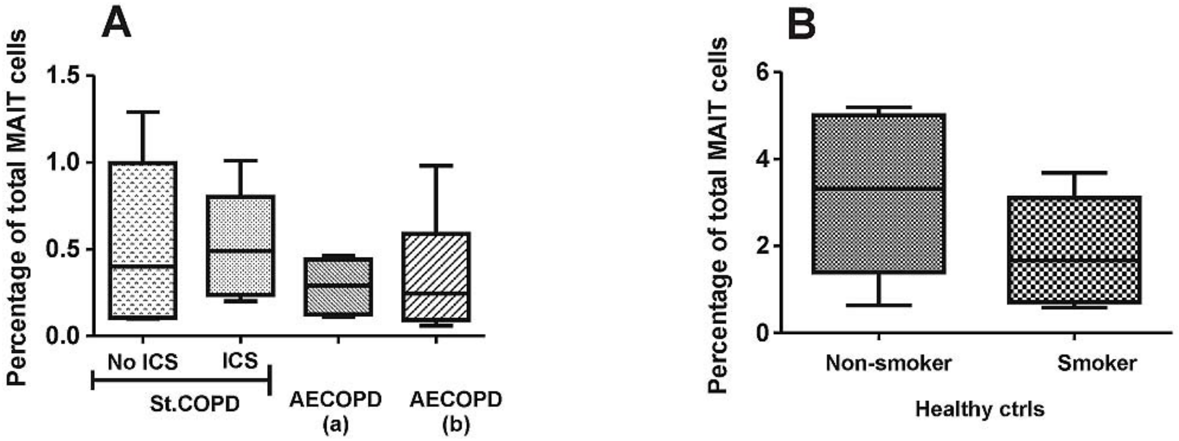

Fig. S4. Percentages of total MAIT cells were evaluated in the peripheral blood of inhaled corticosteroid treated (ICS) or non-treated (No ICS) stable COPD and AECOPD patients (**A**). Data present here were derived from five/six stable COPD patients under no ICS/ICS therapy, and ten AECOPD blood donors all receiving ICS therapy.

Percentages of total MAIT cells were measured in the peripheral blood of non-smoker and smoker populations of healthy controls (**B**). Data present here were derived from eleven non-smoker and five smoker healthy control blood donors. Boxes show interquartile ranges (IQR) whiskers represent lowest and highest values, horizontal lines indicate median. Asterisks represent significant *p* (*< 0.05) values.
